# Improved Vascularization and Survival of White Compared to Brown Adipose Tissue Grafts in the Dorsal Skinfold Chamber

**DOI:** 10.3390/biomedicines10010023

**Published:** 2021-12-23

**Authors:** Andrea Weinzierl, Yves Harder, Daniel Schmauss, Emmanuel Ampofo, Michael D. Menger, Matthias W. Laschke

**Affiliations:** 1 Institute for Clinical and Experimental Surgery, Saarland University, 66421 Homburg, Germany; emmanuel.ampofo@uks.eu (E.A.); michael.menger@uks.eu (M.D.M.); matthias.laschke@uks.eu (M.W.L.); 2 Department of Plastic, Reconstructive and Aesthetic Surgery, Ospedale Regionale di Lugano, Ente Ospedaliero Cantonale (EOC), 6900 Lugano, Switzerland; yves.harder@eoc.ch (Y.H.); Daniel.Schmauss@eoc.ch (D.S.); 3 Faculty of Biomedical Sciences, Università della Svizzera Italiana, 6900 Lugano, Switzerland

**Keywords:** brown adipose tissue, white adipose tissue, fat graft, vascularization, microcirculation, dorsal skinfold chamber, intravital fluorescence microscopy

## Abstract

Fat grafting is a frequently applied procedure in plastic surgery for volume reconstruction. Moreover, the transplantation of white adipose tissue (WAT) and brown adipose tissue (BAT) increasingly gains interest in preclinical research for the treatment of obesity-related metabolic defects. Therefore, we herein directly compared the vascularization capacity and survival of WAT and BAT grafts. For this purpose, size-matched grafts isolated from the inguinal WAT pad and the interscapular BAT depot of C57BL/6N donor mice were syngeneically transplanted into the dorsal skinfold chamber of recipient animals. The vascularization and survival of the grafts were analyzed by means of intravital fluorescence microscopy, histology, and immunohistochemistry over an observation period of 14 days. WAT grafts showed an identical microvascular architecture and functional microvessel density as native WAT. In contrast, BAT grafts developed an erratic microvasculature with a significantly lower functional microvessel density when compared to native BAT. Accordingly, they also contained a markedly lower number of CD31-positive microvessels, which was associated with a massive loss of perilipin-positive adipocytes. These findings indicate that in contrast to WAT grafts, BAT grafts exhibit an impaired vascularization capacity and survival, which may be due to their higher metabolic demand. Hence, future studies should focus on the establishment of strategies to improve the engraftment of transplanted BAT.

## 1. Introduction

In humans, three types of fat tissue consisting of white, brown, and beige/brite adipocytes can be differentiated. White adipose tissue (WAT) is the most prevalent type throughout the body and acts as an energy storage in triglyceride form. In addition, it fulfills other important functions, such as the secretion of inflammatory and metabolic mediators [1]. Brown adipose tissue (BAT) is characterized by a higher number of mitochondria within each cell, permitting the conversion of stored energy into body heat by uncoupling oxidative phosphorylation. In humans, BAT disappears with age and remnants only persist in few locations [2]. The main depots in humans are located in the supraclavicular and neck region as well as paravertebrally, mediastinally, paraaortically, and suprarenally. In contrast, in rodents BAT is located in an interscapular depot [3]. Even though the extent of BAT activity and its influence on the metabolism is more pronounced in rodents, active BAT can be found in a significant number of adult humans [4]. BAT activity in humans is acutely cold induced and is stimulated via the sympathetic nervous system [3]. Interestingly, the quantity of BAT in adults is inversely correlated with the body mass index and is less present in obese patients [5]. Furthermore, obese patients with a high BAT activity have a higher glucose tolerance than patients with low BAT activity, suggesting that the presence of active brown adipocytes promotes metabolic homeostasis [6]. Beige/brite adipocytes are brown-like adipocytes that develop within subcutaneous WAT depots upon cold stimulation or exercise [7,8].

The emerging role of adipose tissue as an endocrine and immunologically active organ with the ability to regulate systemic energy homeostasis suggests its future therapeutic use for the treatment of obesity and associated metabolic diseases [9]. This may be achieved by inducing transient browning of adipocytes using phytochemicals [10,11]. Another highly promising approach is the transplantation of WAT or BAT [12]. The grafting of WAT is aimed at harnessing its beneficial effects regarding lipogenesis, fatty acid oxidation, or adipokine secretion as well as its high capacity to prevent detrimental ectopic lipid storage [13]. For instance, the transplantation of WAT from wild-type donor mice into leptin-deficient ob/ob mice has been shown to normalize their metabolic, immune, and inflammatory alterations and to prevent further body weight gain [14]. Similarly, the transplantation of BAT has been explored to raise energy expenditure, to improve metabolic health or even to counteract certain endocrinopathies, such as polycystic ovary syndrome [15,16,17]. Both, WAT and BAT grafts are crucially dependent on a rapid and sufficient vascularization after transplantation. However, the transplantation of BAT may be more challenging due to the fact that it has a high metabolic demand and, thus, contains much more microvessels when compared to WAT [18,19].

To test this hypothesis in the present study, we directly compared the vascularization capacity and survival of BAT and WAT grafts. For this purpose, we transplanted size-matched grafts isolated from the inguinal WAT pad and the interscapular BAT depot of C57BL/6N donor mice into the dorsal skinfold chamber of recipient animals. The formation of new microvessels and tissue architecture of the grafts were subsequently analyzed by means of intravital fluorescence microscopy, histology, and immunohistochemistry during an observation period of 14 days.

## 2. Materials and Methods

### 2.1. Animals

All animal experiments were approved by the local governmental animal protection committee (permit number: 30/2020, approved 5 October 2020) and conducted in accordance with the European legislation on the protection of animals (Directive 2010/63/EU) and the NIH Guidelines on the Care and Use of Laboratory Animals (NIH publication #85-23 Rev. 1985).

In this study, 5 C57BL/6N mice (Institute for Clinical & Experimental Surgery, Saarland University, Homburg/Saar, Germany) were used for the in vivo imaging of native adipose tissue. The mice were used at an age of up to 52 weeks and a body weight of 28–32 g. Additional 4 C57BL/6N mice of the same age and body weight served as fat donors for the isolation of BAT and WAT grafts. Both types of fat grafts were implanted into the dorsal skinfold chambers of 8 male C57BL/6N mice with an age of 12–20 weeks and a body weight of 23–26 g. The animals were kept at a room temperature of 22–24 °C and a 12 h day–night cycle. They had free access to standard pellet chow (Altromin, Lage, Germany) and tap water. Mice with a dorsal skinfold chamber were kept one per cage for the duration of the experiments.

### 2.2. Anesthesia

For the harvesting of adipose tissue, the implantation of the dorsal skinfold chamber and intravital fluorescence microscopic analyses, the animals were anesthetized by injecting ketamine (100 mg/kg body weight; Ursotamin^®^; Serumwerke Bernburg, Bernburg, Germany) and xylazine (12 mg/kg body weight; Rompun^®^; Bayer, Leverkusen, Germany) intraperitoneally. In addition, they received a subcutaneous injection of 5 mg/kg carprofen (Rimadyl^®^; Zoetis Deutschland GmbH, Berlin, Germany) for perioperative analgesia.

### 2.3. Dorsal Skinfold Chamber Model and Fat Grafting

**Figure 1:**
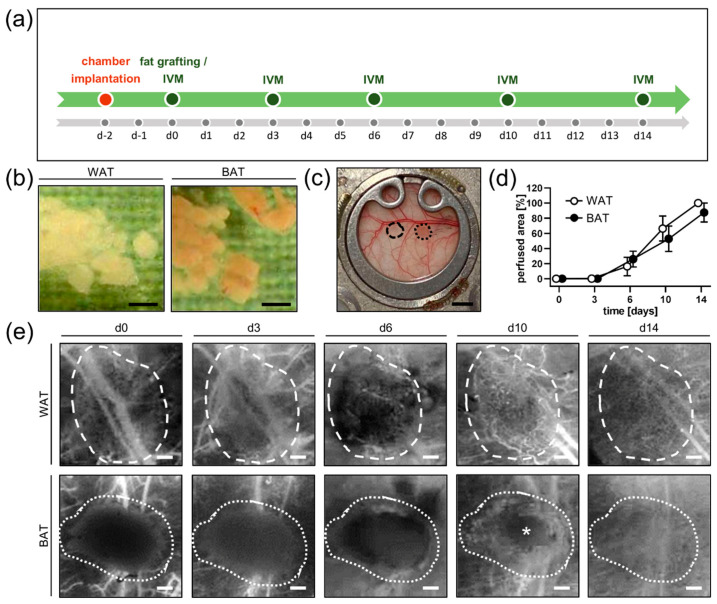
(**a**) Study design of the performed experiments. The implantation of the dorsal skinfold chamber was executed 48 h prior to fat grafting into C57BL/6N mice (*n* = 8). Graft vascularization was repeatedly analyzed by means of intravital fluorescence microscopy (IVM) on day 0, 3, 6, 10, and 14 after transplantation. (**b**) WAT and BAT samples excised from C57BL/6N donor mice and minced for grafting. Scale bar: 500 μm. (**c**) Overview of the observation window of a dorsal skinfold chamber with a WAT (broken line) and a BAT (dotted line) graft directly after transplantation. Scale bar: 2 mm. (**d**) Perfused area of WAT (*n* = 8) and BAT (*n* = 8) grafts on day 0, 3, 6, 10, and 14 after transplantation, as assessed by intravital fluorescence microscopy. Mean  ±  SEM. (**e**) Representative intravital fluorescence microscopic images (blue light epi-illumination with 5% FITC-labeled dextran for contrast enhancement) of a WAT (broken line) and a BAT (dotted line) graft over the course of the 14-day observation period. Note that the center of the BAT graft still contains a non-vascularized area (asterisk) on day 10, whereas the WAT graft is already fully vascularized at this time point. Scale bar: 200 μm.

A dorsal skinfold chamber was implanted into C57BL/6N mice, as described previously in detail [20]. In brief, the depilated dorsal skinfold was extended and sandwiched between two titanium chamber frames (Irola Industriekomponenten GmbH & Co. KG, Schonach, Germany). One layer of skin was completely removed in a circular area of ~15 mm in diameter. The remaining layers of striated panniculus carnosus muscle, subcutaneous tissue and skin served as the host tissue for fat grafts. The chamber was sealed with a removable cover glass and snap ring. Thereafter, the mice could recover for 48 h to exclude alterations of the microcirculation due to anesthesia or surgical trauma (Figure 1a).

The interscapular BAT and the inguinal subcutaneous WAT of euthanized donor mice were excised, taking care not to contain the inguinal lymph node. The tissue was then minced by means of a histology tissue cutter (McIlwain Tissue Chopper, CLE Co., Ltd., Gomshall, UK) to produce fat grafts of an identical volume (0.5 × 0.5 × 0.5 mm), which were rinsed in 0.9% NaCl solution (Figure 1b). Subsequently, the cover glass of the chamber window of the anesthetized recipient mice was removed and one BAT and one WAT graft were placed into each chamber (Figure 1c). Thereafter, the chamber was resealed with a new cover glass and the snap ring. Intravital fluorescence microscopy of the fat grafts was performed directly after transplantation, i.e., day 0, as well as on days 3, 6, 10, and 14. After the last microscopy, the animals were euthanized by cervical dislocation and the dorsal skinfold chamber preparations were carefully excised for further histological and immunohistochemical analyses. All animals tolerated the chamber implantation and fat grafts well and showed normal feeding and sleeping habits during the observation period.

### 2.4. Preparation of Native BAT and WAT

To analyze the vascularization of BAT and WAT in situ, intravital fluorescence microscopy was performed on surgically exposed adipose tissue. For this purpose, the dorsal skin of the anesthetized animals was excised over the scapular region. The deep interscapular BAT was exposed unilaterally by microsurgical blunt dissection of the overlying superficial WAT, which was then excised. Both, the liberated BAT and the contralateral superficial WAT were then protected from desiccation with a cover glass and intravital fluorescence microscopy was performed. Thereafter, the animals were killed by cervical dislocation and WAT and BAT tissue samples were excised for further histological and immunohistochemical analyses.

### 2.5. Intravital Fluorescence Microscopy

For intravital fluorescence microscopy, the anesthetized animals were fixed on a plexiglass platform and received 0.1 mL of the blood plasma marker 5% fluorescein isothiocyanate (FITC)-labeled dextran (150,000 Da; Sigma-Aldrich, Taufkirchen, Germany) for contrast enhancement by intravenous retrobulbar injection. The dorsal skinfold chamber or the in situ exposed adipose tissue were then placed under a Zeiss Axiotech fluorescence epi-illumination microscope (Zeiss, Oberkochen, Germany) and the microscopic images were recorded with a charge-coupled device video camera (FK6990; Pieper, Schwerte, Germany) and a DVD system. Microscopies were performed at a constant room temperature of ~22 °C. In addition to a panoramic view, four peripheral and one central regions of interest (ROI), each consisting of 4 high-power fields (HPF), or 5 ROI per native adipose tissue were recorded for further analyses.

The microscopic images were analyzed by means of the offline analysis system CapImage (Version 8.5, Zeintl, Heidelberg, Germany). The analysis included the quantitative assessment of the perfused graft area (in % of the total graft area). Moreover, the functional microvessel density (FMD), i.e., the overall length of all red blood cell (RBC)-perfused microvessels per observation area (in cm/cm^2^) was determined. In addition, microhemodynamic parameters were measured in up to 5 randomly chosen microvessels per ROI, as soon as blood perfusion could be detected. Vessel diameters (D, in µm) were measured perpendicular to the vessel path. The centerline RBC velocity (V, in µm/s) was assessed using the line shift method [21]. The volumetric blood flow (VQ, in pL/s) was calculated from V and D as VQ = π × (D/2)^2^ × V/K where K (= 1.6) represents the Baker–Wayland factor considering the parabolic velocity profile of blood in microvessels [22]. Additionally, the measured microhemodynamic parameters were used to calculate the wall shear rate (y, in s^−1^) by means of the Newtonian definition y = 8 × V/D.

### 2.6. Histology and Immunohistochemistry

Tissue samples were fixed in formalin, embedded in paraffin, and cut into 3 µm thick sections. Hematoxylin and eosin (HE) stainings of individual sections were performed according to standard procedures. The sections were subsequently assessed using a BX60 microscope (Olympus, Hamburg, Germany) and the imaging software cellSens Dimension 1.11 (Olympus).

For the immunohistochemical detection of adipocytes, tissue sections were stained with a monoclonal rabbit anti-mouse antibody against perilipin (1:200; Cell Signaling Technology, Danvers, MA, USA) as primary antibody. Additional sections for the detection of brown adipocytes were stained with a polyclonal rabbit anti-mouse antibody against the BAT-specific uncoupling protein (UCP)-1 (1:100, Abcam, Cambridge, UK) as primary antibody. A goat anti-rabbit peroxidase-labeled antibody (1:100; Jackson ImmunoResearch Laboratories, West Grove, PA, USA) served as secondary antibody. The used chromogen was 3-amino-9-ethylcarbazole (Abcam). All sections were counterstained with Mayer’s hemalum (Merck, Darmstadt, Germany).

For the immunohistochemical detection of endothelial cells, tissue sections were stained with a monoclonal rat anti-mouse antibody against CD31 (1:100; Dianova, Hamburg, Germany) as primary antibody and a goat anti-rat Alexa 555 antibody (1:100; Invitrogen, Waltham, MA, USA) as secondary antibody. On each section, cell nuclei were stained with Hoechst 33342 (2 µL/mL; Sigma-Aldrich) to merge images exactly. The sections were used to assess the density of CD31+ microvessels (given in mm-2) within 2 HPF per graft or native tissue sample.

### 2.7. Statistical Analysis

After testing the data for normal distribution and equal variance, differences between two groups were analyzed by the unpaired Student’s *t*-test (GraphPad Prism 9; GraphPad Software, San Diego, CA, USA). In case of non-parametric data, a Mann–Whitney rank sum test was used. In case of the comparison of three groups, a one-way ANOVA with Tukey’s multiple comparisons test as a post hoc analysis was used. All values are expressed as means  ±  standard error of the mean (SEM). Statistical significance was accepted for a value of *p*  <  0.05.

## 3. Results

### 3.1. Intravital Fluorescence Microscopy

The vascularization of WAT and BAT grafts within the dorsal skinfold chamber of recipient mice was analyzed by means of repeated intravital fluorescence microscopy. First, blood-perfused microvessels could be observed in both graft types on day 6 after transplantation. Throughout the following days, the perfused area of the grafts progressively increased without marked differences between the two groups. Except for one BAT transplant that failed to engraft, all fat grafts were completely vascularized by day 14 (Figure 1d,e).

**Figure 2:**
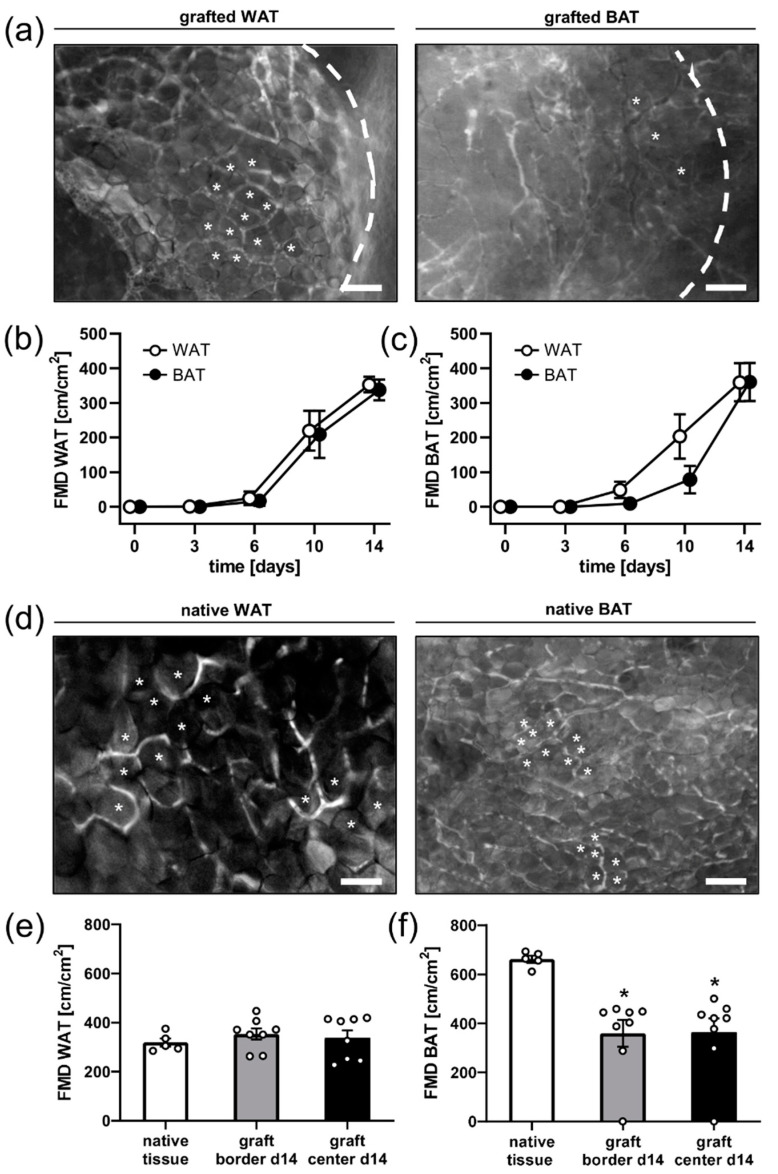
(**a**) Representative intravital fluorescence microscopic images (blue light epi-illumination with 5% FITC-labeled dextran for contrast enhancement) of a WAT and a BAT graft on day 14 after transplantation (broken lines = graft borders). Note that the WAT graft presents as homogenous adipose tissue, which typically consists of a dense network of microvessels surrounding individual adipocytes (asterisks) in a regular fashion. BAT grafts are characterized by a chaotic vessel architecture and only a few clearly identifiable adipocytes (asterisks). Scale bar: 50 μm. (**b**,**c**) FMD in the border (*n* = 8) and center (*n* = 8) of WAT grafts (**b**) and BAT grafts (**c**) on day 0, 3, 6, 10, and 14 after transplantation, as assessed by intravital fluorescence microscopy. Mean  ±  SEM. (**d**) Representative intravital fluorescence microscopic images (blue light epi-illumination with 5% FITC-labeled dextran for contrast enhancement) of native WAT and BAT. Both tissues display a network of microvessels surrounding individual adipocytes (asterisks) in a regular fashion. The density of these microvessels is markedly higher in native BAT compared to WAT. Scale bar: 50 μm. (**e**,**f**) FMD of native tissue (white bars; *n* = 5) as well as the border (grey bars; *n* = 8) and center (black bars; *n* = 8) of WAT (**e**) and BAT (**f**) grafts on day 14 after transplantation, as assessed by means of intravital fluorescence microscopy. Mean  ±  SEM; * *p * <  0.05 vs. native tissue.

The FMD of WAT grafts did not significantly differ between the analyzed border and center regions (Figure 2a,b). In contrast, the vascularization of the center of BAT grafts was delayed, as indicated by a lower FMD between days 6 and 10 after transplantation when compared to that of the border regions (Figure 2a,c). On day 14, both WAT and BAT grafts exhibited a FMD of ~350 cm/cm^2^ (Figure 2b,c). However, their final microvascular architecture markedly differed. In fact, BAT grafts were characterized by a chaotic architecture without a recognizable orientation or organization of microvessels and only a few clearly identifiable adipocytes (Figure 2a). WAT grafts presented as homogenous adipose tissue (Figure 2a), which typically consists of a dense network of microvessels surrounding individual adipocytes in a regular fashion. This phenotypic difference became even more apparent when further comparing the vessel architecture of WAT and BAT grafts with their corresponding native counterparts (Figure 2a,d). Of interest, native BAT exhibited a FMD of almost 700 cm/cm^2^, which was twofold of that measured in native WAT (350 cm/cm^2^). Comparisons between grafts and native tissues showed that WAT grafts and native WAT exhibit a comparable physiological architecture and FMD (Figure 2a,d,e). In contrast, BAT grafts presented with a massively altered tissue architecture and a significantly lower final FMD when compared to native BAT (Figure 2a,d,f).

The analysis of microhemodynamic parameters revealed a progressive decrease in the diameter of individual microvessels within the border and center regions of both WAT and BAT grafts over time without significant differences between the two groups (Table 1). At the end of the observation period, the microvessel diameters of the grafts were slightly larger when compared to native WAT and BAT. The microvessels within WAT grafts exhibited a constant centerline RBC velocity between day 6 and 14, which was comparable to that of microvessels within native WAT (Table 1). In contrast, the centerline RBC velocity of microvessels within BAT grafts was initially very low, but progressively increased until the end of the in vivo observation period. Accordingly, calculated values of the volumetric blood flow and shear rate were also higher in WAT grafts when compared to BAT grafts (Table 1). In both graft types, these values were still significantly lower on day 14 after transplantation when compared to native WAT and BAT.

### 3.2. Histology and Immunohistochemistry

**Figure 3:**
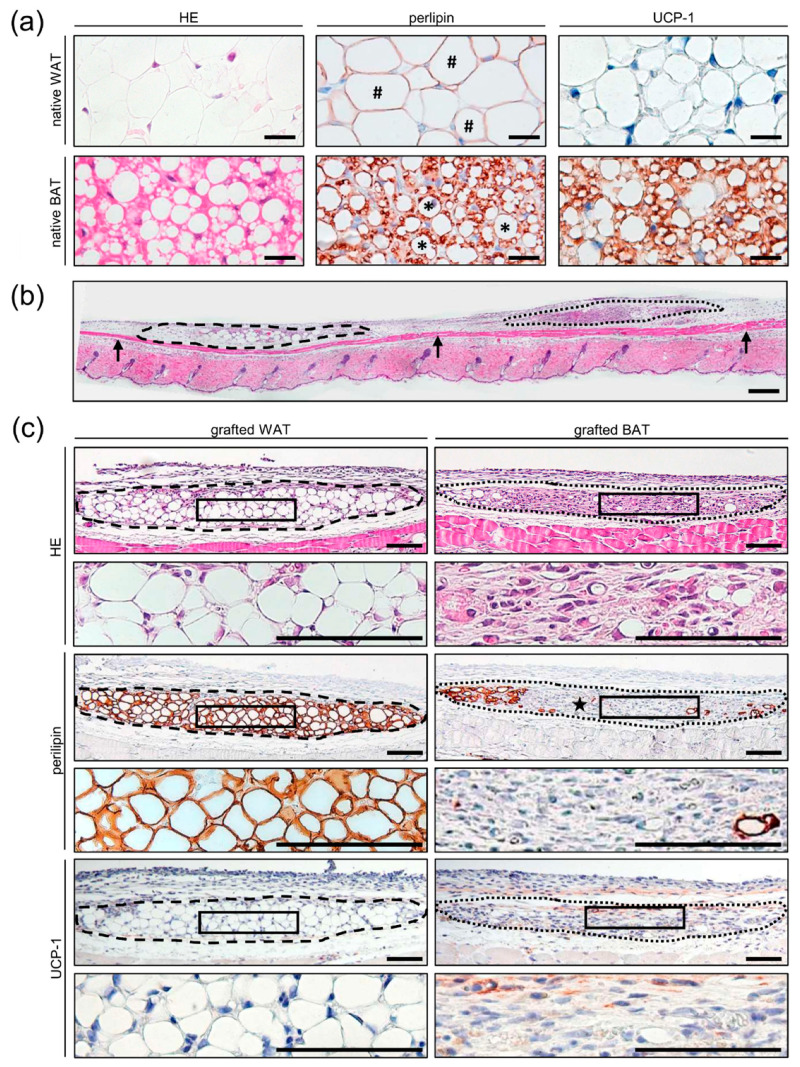
(**a**) HE-, perilipin-, and UCP-1-stained sections of native WAT and BAT. Note that native WAT consists of large white adipocytes (hashtags) with central fat reservoirs and peripheral nuclei. Native BAT is characterized by a high density of small brown adipocytes (asterisks) containing multiple lipid droplets and a dense, mitochondria-rich cytoplasm. In contrast to native BAT, no UCP-1 signal is detected in native WAT. Scale bars: 10 μm (**b**) HE-stained section of a dorsal skinfold chamber preparation with a WAT graft (border marked by broken line) and a BAT graft (border marked by dotted line) located on top of the panniculus carnosus muscle (arrows) on day 14 after transplantation. Scale bar: 100 μm. (**c**) HE-, perilipin-, and UCP-1-stained WAT and BAT graft sections on day 14 after transplantation. Note that the WAT graft exhibits an intact tissue architecture, whereas the BAT graft contains large areas of unspecific granulation tissue (star) in its center and only a few remaining perilipin-positive adipocytes in its border regions. Moreover, the BAT graft no longer exhibits a ubiquitous UCP-1 signal as a clear sign of brown adipocyte regression. Higher magnifications of inserts (border marked with black line) beneath the respective images. Scale bars: 50 μm.

The cellular composition and morphology of native WAT and BAT were analyzed by means of histology and immunohistochemistry. HE- and perilipin-stained sections revealed that native WAT consists of large white adipocytes with central fat reservoirs and peripheral nuclei (Figure 3a). In contrast, native BAT was characterized by a high density of small brown adipocytes containing multiple lipid droplets and a dense, mitochondria-rich cytoplasm (Figure 3a). Furthermore, native BAT abundantly expressed UCP-1, while this protein was absent in white adipocytes (Figure 3a).

Both WAT and BAT grafts could be clearly identified on top of the panniculus carnosus muscle layer on HE-stained sections of excised dorsal skinfold chamber preparations on day 14 after fat transplantation (Figure 3b,c). Additional perilipin-stained tissue sections revealed an intact tissue architecture of WAT grafts without marked structural differences when compared to native WAT (Figure 3a,c). In contrast, BAT grafts exhibited large areas of unspecific granulation tissue in their center and only a few remaining perilipin-positive adipocytes in their border regions (Figure 3c). Additional UCP-1-stained sections showed clear signs of BAT regression, as the grafted BAT no longer exhibited a ubiquitous UCP-1 signal (Figure 3c).

**Figure 4:**
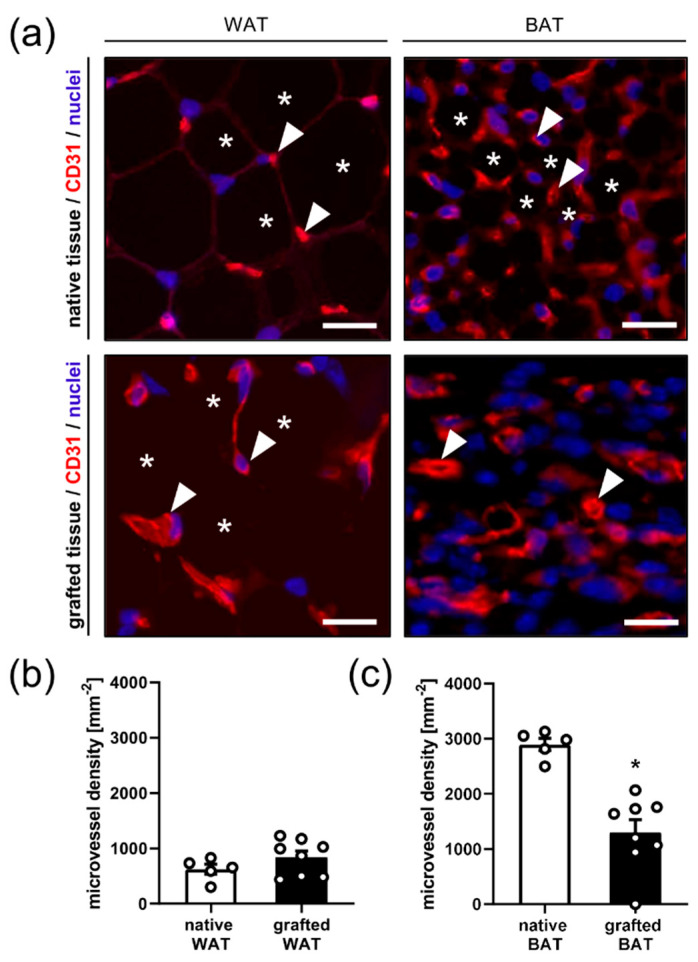
(**a**) Immunofluorescent CD31-stained sections of native WAT and BAT as well as WAT and BAT grafts on day 14 after transplantation. Cell nuclei were stained with Hoechst 33342. Note that native and grafted WAT shows regularly arranged microvessels (arrowheads) between large white adipocytes (asterisks). Native BAT exhibits a regular pattern of small brown adipocytes (asterisks) surrounded by microvessels (arrowheads), whereas grafted BAT consists of a chaotic microvascular network without clearly identifiable brown adipocytes. Scale bars: 20 μm. (**b**,**c**) Microvessel density of native tissue (white bars; *n* = 5) and grafted WAT (**b**); black bar; *n* = 8) and BAT (**c**); black bar; *n* = 8) on day 14 after transplantation, as assessed by means of immunohistochemistry. Mean  ±  SEM; * *p*  <  0.05 vs. native tissue.

Additional immunohistochemical analyses served for the measurement of the density of CD31-positive microvessels in native and grafted WAT and BAT (Figure 4). Native and grafted WAT were characterized by regularly arranged individual microvessels between large white adipocytes without significant differences in microvessel density (Figure 4a,b). Native BAT presented with a regular pattern of small brown adipocytes surrounded by microvessels, which exhibited a markedly higher density when compared to native and grafted WAT (Figure 4a–c). In contrast, grafted BAT consisted of a chaotic microvascular network without clearly identifiable brown adipocytes in between and a significantly lower microvessel density when compared to native BAT (Figure 4a,c).

## 4. Discussion

WAT grafting is widely used for breast reconstruction after tumor excision. However, the safety of this procedure is questionable, because WAT is a rich source of endothelial progenitor cells and, thus, may markedly contribute to the formation and vascularization of new breast cancer lesions [23,24,25]. Due to their positive impact on metabolism, fat grafts have additionally emerged over the last decades as an innovative treatment option for obesity-related metabolic pathologies [12]. Contrary to WAT grafts, which mainly improve glucose tolerance or insulin sensitivity, BAT grafts may also aid in decreasing excessive fat storage and targeting obesity directly by increasing the organism’s energy expenditure and heat production [15,16,26]. Native BAT is highly blood perfused to provide brown adipocytes with the necessary resources for thermogenesis [27]. The dense vascularization of WAT, in turn, promotes the major function of its white adipocytes to store and release energy in the form of triglycerides [28]. We therefore hypothesized in the present study that the success of WAT and BAT transplantation is crucially determined by a rapid and sufficient vascularization of the grafts. In line with this view, we found that both types of grafts completely vascularized within the first 14 days after transplantation. However, only WAT grafts developed blood-perfused networks with a physiological pattern and an FMD comparable to that of native WAT. In contrast, microvessels in BAT grafts formed chaotic networks, which did not grow fast enough into the central graft regions and exhibited a markedly lower FMD when compared to native BAT. Accordingly, major parts of these grafts regressed over time. For our experiments we used the well-established dorsal skinfold chamber model, because it allows the non-invasive in vivo analysis of angiogenesis and vascular remodeling within grafts and their surrounding host tissue throughout an observation period of 2–3 weeks [29]. In combination with the technique of intravital fluorescence microscopy, it is thereby possible to quantitatively assess morphological and microhemodynamic parameters of newly developing microvascular networks. Of interest, this model has also been used in previous studies to study the vascularization of fat grafts [30,31]. Langer et al. [30] showed in a proof-of-concept study that WAT grafts vascularize well within the chamber. Thus, they concluded that this model is suitable to study the physiology of adipose tissue revascularization. In line with this study, we could demonstrate that WAT grafts are fully blood-perfused on day 14 after transplantation and exhibit a physiological adipose tissue architecture.

To our knowledge, the engraftment of BAT has not yet been analyzed in the dorsal skinfold chamber model. However, several studies have investigated the performance of BAT grafts using subcutaneous tissue as the transplantation site. Chen et al. [32] analyzed whether both WAT and BAT grafts are capable of restoring the diabetes-mediated inhibition of ischemia-induced angiogenesis in a high-fat diet (HFD) mouse model of hindlimb ischemia. Of interest, they found that subcutaneous WAT grafts ameliorate HFD-induced insulin resistance and markedly reduce the release of several proinflammatory cytokines, while BAT grafts fail to improve insulin sensitivity. Similarly, Stanford et al. [33] reported that subcutaneous BAT grafts do not improve glucose tolerance, which can be overcome by their transplantation into the abdominal cavity. These findings may be explained by a relatively low microvascular density of subcutaneous tissue, which is not sufficient to adequately vascularize BAT grafts before tissue degeneration and necrosis occurs. In the present study, BAT grafts were transplanted onto highly vascularized striated muscle tissue. Nonetheless, they still did not develop a microvasculature with a FMD comparable to native BAT. This indicates that even within the well-perfused host tissue environment of the dorsal skinfold chamber, BAT grafts fail to engraft successfully, most probably due to their high metabolic demand.

Our repeated intravital fluorescent microscopic analyses revealed completely different vascularization patterns of WAT and BAT grafts over time. In fact, WAT grafts rapidly developed a highly organized microvasculature, which was comparable to that of native WAT. In contrast, BAT grafts finally exhibited a non-physiological, chaotic architecture without a recognizable orientation or organization of microvessels. This indicates that the vascularization mode of WAT grafts may be mainly based on inosculation, i.e., the interconnection of pre-existing microvessels within the graft tissue with the host microvasculature of the transplantation site [34]. In line with this view, the microvessels within WAT grafts exhibited constantly high centerline RBC velocities immediately after the onset of blood perfusion on day 6 until day 14, which were comparable to those of microvessels within native WAT. On the other hand, BAT grafts may be mainly vascularized via the process of sprouting angiogenesis, i.e., the progressive ingrowth of newly formed microvessels from the surrounding host tissue [35]. This vascularization mode is typically associated with an initially low but then increasing centerline RBC velocity, volumetric blood flow, and shear rate of individual microvessels over time [36]. Accordingly, differences in these microhemodynamic parameters were particularly pronounced between WAT and BAT grafts in the early phase of graft vascularization on day 6. Moreover, it is well known that the diameter of newly formed microvessels continuously decreases during the process of vessel maturation [37,38]. A reduction in microvessel diameters could be observed in both WAT and BAT grafts. However, in WAT grafts the initially large microvessel diameters may rather be interpreted as the dilation of pre-existing microvessels due to an insufficient early blood drainage after inosculation than as a typical sign of newly formed, immature microvessels during angiogenesis.

In addition, we could demonstrate a clear negative correlation between the extent of vascularization and the regression of adipose tissue grafts. In this context, our novel data indicate that strategies are particularly required to improve the vascularization of BAT grafts. These strategies may include the application of pharmacological compounds, such as angiogenic growth factors [39]. It may also be promising to combine fat grafting with cellular tissue engineering concepts for vascularization, including the co-transplantation with adipose tissue-derived stem cell-rich stromal vascular fraction [40,41].

A possible limitation of this study is its mainly descriptive character with a focus on morphological changes in grafted WAT and BAT. Hence, it may be interesting to additionally clarify the effects of these grafts on tissue metabolism in future studies. Furthermore, it should be considered that the findings obtained in the used murine model may not be entirely transferable to human conditions. In fact, in rodents BAT is crucial for the maintenance of energy balance, while in humans, this function is less consistent [42].

In conclusion, the present study demonstrates that WAT successfully engrafts after transplantation into the dorsal skinfold chamber of mice and rapidly establishes a blood-perfused microvasculature with a physiological network morphology and microvessel density. In contrast, BAT grafts do not reach a physiological microvessel density and regress over time. Accordingly, their impaired vascularization cannot cover their higher metabolic demand. Hence, future studies should focus on the establishment of vascularization strategies to improve the survival and approaches in the treatment of metabolic pathologies and obesity.

## Figures and Tables

**Table 1:** Diameter, centerline RBC velocity, volumetric blood flow, and shear rate of microvessels within native WAT and BAT tissue (*n* = 5) as well as within the border (*n* = 8) and the center (*n* = 8) of WAT and BAT grafts on day 0, 3, 6, 10, and 14 after transplantation, as assessed by means of intravital fluorescence microscopy.

	d0	d3	d6	d10	d14	Native Tissue
**Diameter (μm)**						
WAT graft border	-	-	16 ± 2	15 ± 1	12 ± 1 *	
WAT graft center	-	-	20 ± 6	16 ± 2	11 ± 1	
						9 ± 0
BAT graft border	-	-	16 ± 0	14 ± 0	12 ± 1	
BAT graft center	-	-	14 ± 3	14 ± 1	11 ± 2	
						8 ± 0
**Centerline RBC velocity (μm/s)**						
WAT graft border	-	-	202 ± 56	186 ± 34	193 ± 23	
WAT graft center	-	-	253 ± 69	188 ± 39	200 ± 18	
						245 ± 11
BAT graft border	-	-	36 ± 11 ^#^	144 ± 13	181 ± 7	
BAT graft center	-	-	20 ± 19	99 ± 30	161 ± 26	
						229 ± 12
**Volumetric blood flow (pL/s)**						
WAT graft border	-	-	31 ± 11	20 ± 3	16 ± 3	
WAT graft center	-	-	53 ± 8	19 ± 4	13 ± 2	
						9 ± 1
BAT graft border	-	-	6 ± 3 ^#^	17 ± 3	17 ± 3 *	
BAT graft center	-	-	4 ± 4 ^#^	9 ± 2	9 ± 1 *^,#^	
						7 ± 1
**Shear rate (s^−1^)**						
WAT graft border	-	-	99 ± 29	109 ± 23	135 ± 16 *	
WAT graft center	-	-	111 ± 54	113 ± 26	148 ± 17 *	
						238 ± 12
BAT graft border	-	-	17 ± 4 ^#^	85 ± 5	130 ± 11 *	
BAT graft center	-	-	7 ± 7	65 ± 23	138 ± 26 *	
						240 ± 14

Mean ± SEM. * *p* < 0.05 d14 grafts vs. native tissue; ^#^ *p* < 0.05 vs. WAT.

## Data Availability

All data can be obtained in this manuscript.
